# Impact of COVID-19 on Incident Depression and Anxiety: A Population-Based Observational Study Using Statewide Claims Data

**DOI:** 10.3390/healthcare13141638

**Published:** 2025-07-08

**Authors:** Jaewhan Kim, Khanh N. C. Duong, Emeka Elvis Duru, Rachel Weir, Karen Manotas, Kristi Kleinschmit, Aaron Fischer, Peter Weir, Fernando A. Wilson

**Affiliations:** 1 Department of Physical Therapy, University of Utah, 520 Wakara Way, Salt Lake City, UT 84108, USA; 2 Department of Pharmacotherapy, University of Utah, 30 South 2000 East, Salt Lake City, UT 84112, USA; khanh.duong@utah.edu (K.N.C.D.); elvis.duru@utah.edu (E.E.D.); 3 Department of Psychiatry, University of Utah, 501 Chipeta Way, Salt Lake City, UT 84108, USA; rachel.weir@hsc.utah.edu (R.W.); karen.manotas@hsc.utah.edu (K.M.); kristi.kleinschmit@hsc.utah.edu (K.K.); 4 Department of Educational Psychology, University of Utah, 1721 Campus Center Drive, Salt Lake City, UT 84112, USA; aaron.fischer@utah.edu; 5 Medical Group Population Health, University of Utah, 50 North Medical Drive, Salt Lake City, UT 84132, USA; peter.weir@utah.edu; 6 Department of Population Health Sciences, University of Utah, 295 Chipeta Way, Salt Lake City, UT 84108, USA; fernando.wilson@utah.edu

**Keywords:** COVID-19, depression, anxiety, all payers claims database, entropy balancing

## Abstract

**Objectives**: Evidence suggests that COVID-19 infection contributes to elevated risks of psychiatric disorders, including depression and anxiety, however, this association remains underexplored. This study aimed to examine the incidence of depression and anxiety in individuals with COVID-19 compared to those without any infection. **Method**: Using the Utah All Payers Claims Database (2019 to 2021), we examined adult patients with continuous insurance enrollment. Individuals with pre-existing depression or anxiety were excluded. COVID-19 infection in 2020 was identified using diagnostic and procedural codes. The Least Absolute Shrinkage and Selection Operator (LASSO) method was applied to select covariates, followed by entropy balancing to adjust for baseline differences. Weighted logistic regression models were used to estimate the association between COVID-19 infection and incident mental health diagnoses in 2021. **Results**: Among 356,985 adults included in the final analytic sample for depression analysis, 37.6 percent had a documented COVID-19 infection in 2020. Individuals with prior infection had significantly higher odds of receiving a depression diagnosis in 2021 compared to those without infection (OR = 1.48, *p* < 0.01). A similar pattern was observed for anxiety: among 371,491 adults, 38.1 percent had a COVID-19 infection, and infected individuals had 46 percent greater odds of receiving an anxiety diagnosis (OR = 1.46, *p* < 0.01), after adjusting for demographic and clinical characteristics. **Conclusions**: This study highlights the elevated risk of depression and anxiety among patients who had been infected with COVID-19, emphasizing the importance of addressing the mental health needs of individuals affected by the virus.

## 1. Introduction

The Coronavirus Disease 2019 (COVID-19) pandemic has had a profound impact on mental health globally [1,2]. Estimates suggest that the global prevalence of depression and anxiety symptoms during the pandemic ranged from about 20 to 35 percent [1]. In the United States, the prevalence of depressive symptoms was more than three times higher during the pandemic compared to pre-pandemic levels, with disproportionate burdens observed among individuals with limited socioeconomic resources and greater exposure to stressors such as job loss and housing insecurity [3]. Contributing factors to this surge in mental health disorders included fear of infection, enforced social isolation, financial instability, bereavement, and persistent uncertainty regarding the pandemic’s long-term effects [4,5,6].

Beyond these population-wide psychosocial stressors, some evidence suggests that the COVID-19 infection itself may lead to neuropsychiatric sequelae. Coronaviruses are known to induce a robust systemic inflammatory response, characterized by elevated levels of cytokines and chemokines that can cross the blood–brain barrier and provoke neuroinflammation, an established pathophysiological mechanism in the development of mood and anxiety disorders [7,8]. Depression and anxiety are not only distressing in their own right, but they are also associated with systemic physiological consequences. These conditions may promote a chronic pro-inflammatory state [9,10,11,12,13,14,15], thereby elevating the risk for cardiometabolic disorders such as cardiovascular disease and diabetes [13]. Individuals with depression or anxiety also face higher rates of chronic pain, poor dietary behaviors [9,11], reduced physical activity [12], and greater susceptibility to maladaptive coping strategies such as illicit substance use [10]. Additionally, infection-specific factors such as social stigma, prolonged “long COVID” symptoms, and the psychological toll of managing with a novel and often unpredictable illness may further heighten vulnerability to psychiatric disorders [5].

Prior studies have reported an elevated risk of psychiatric conditions following COVID-19 infection, independent of broader pandemic stressors [3,15,16]. For example, a cohort study found that individuals with COVID-19 were 60 percent more likely to develop mental health disorders compared to uninfected individuals [17]. However, many of these studies rely on self-reported symptoms, cross-sectional surveys, or electronic health records from limited healthcare systems, which may underrepresent clinically diagnosed outcomes and confounding factors. Additionally, while some global health authorities such as the World Health Organization have issued broad statements on the mental health burden of COVID-19 [2], population-level estimates based on rigorous administrative claims data remain sparse. Building on this foundational work, the current study utilizes a comprehensive statewide claims database to examine the association between COVID-19 infection and the risk of depression and anxiety among adults.

## 2. Materials and Methods

Study Design and Data: This retrospective cohort study utilized data from the Utah All Payers Claims Database (APCD) from 2019 to 2021. The Utah APCD includes medical and pharmacy claims, as well as enrollment information, for approximately 70 percent of insured individuals in Utah, including individuals covered by private insurance, Medicaid, and Medicare Advantage [16,18,19]. For this analysis, only data from private insurance and Medicaid were used. The APCD consists of enrollment and claims files. Enrollment data provide demographic and coverage information, including insurance type, coverage dates, age, sex, and race/ethnicity. Claims data capture medical, pharmacy, and dental service records, including service dates, place of service, provider specialty, diagnosis codes—International Classification of Diseases, Tenth Revision (ICD-10) and procedure codes—Current Procedure Terminology (CPT), as well as medication data such as National Drug Codes, NDC and drug names. This study was approved by Institutional Review Board (IRB) at the University of Utah (IRB 00151091).

Study Population: The study cohort included adults aged 18 to 62 years with continuous enrollment in either private insurance or Medicaid from 2019 through 2021. This age range was selected to represent adults covered by private insurance and Medicaid, and to minimize confounding due to Medicare enrollment, which is not comprehensively captured in the Utah APCD. This restriction ensures uniform insurance exposure and improves the interpretability of comparisons across demographic subgroups. Individuals dually eligible for Medicare and Medicaid were excluded. For each outcome (depression or anxiety), we excluded individuals with documented diagnoses of the respective condition in 2019 or 2020, ensuring assessment of incident mental health diagnoses occurring in 2021.

Exposure Variable: The primary independent variable was a diagnosed COVID-19 infection in 2020, identified using a combination of ICD-10 diagnosis codes (J1281, J1282, U071, U099, B948, B9729, Z8616, O985, Z20828); and CPT codes associated with COVID-19 testing and diagnosis (86413, 86328, 86769, 87426, 87428, 87635, 87636, 87637, 87811, 87913, C9803) [20,21]. These codes capture clinically confirmed COVID-19 infections documented within healthcare encounters. Cases without formal diagnostic or testing codes, including suspected or asymptomatic infections not recorded in claims, were not included.

Outcome Measures: The primary outcomes were incident diagnoses of depression and anxiety occurring in 2021. These were identified using ICD-10 codes defined by the Centers for Medicare and Medicaid Services (CMS) and the Agency for Healthcare Research and Quality (AHRQ) Clinical Classification Software Refined (CCSR-v2024.1) [22,23]. Diagnoses were captured from both inpatient and outpatient claims.

Covariates: Covariates included age in 2019, sex, race/ethnicity (categorized as non-Hispanic White, non-Hispanic Other, Hispanic, or unknown), Medicaid enrollment status (yes/no), and comorbidity burden. Comorbidity was measured using the Elixhauser Comorbidity Index, which aggregates diagnostic data from 2019 to generate a weighted comorbidity severity score. In addition to the composite index, individual physical and mental health conditions such as hypertension, diabetes, cognitive disorders, ADHD, personality disorders, schizophrenia, and bipolar disorder were identified through ICD-10 codes using CMS and AHRQ CCSR classification [22,24].

Statistical Analysis: Descriptive statistics were used to summarize baseline characteristics of the study population. Continuous variables were reported as means and standard deviations (SD), while categorical variables were presented as frequencies and percentages. Group comparisons between individuals with and without documented COVID-19 infection in 2020 were conducted using *t*-tests for continuous variables and chi-square tests for categorical variables. The Least Absolute Shrinkage and Selection Operator (LASSO) approach was utilized to select covariates for inclusion in the regression. LASSO is a regularization method that applies a penalty to regression coefficients, shrinking less informative variables toward zero and thereby selecting the most relevant predictors. This improves model interpretability and reduces overfitting, especially in high-dimensional datasets with many potential covariates. A 10-fold Cross-validation procedure (CV) was applied to determine the optimal penalty term (lambda). The selected lambda value (0.0002) was the same for both depression and anxiety models, suggesting a low risk of underfitting. Out of 35 candidate variables, LASSO retained 19 predictors for the depression model, and 23 the anxiety outcome model.

To reduce confounding and ensure comparability between groups, Entropy Balancing (EB) was applied to reweight individuals with and without COVID-19 infection. Entropy balancing is a multivariate reweighting method used to achieve covariate balance between groups by aligning the first three statistical moments (mean, variance, and skewness) of selected covariates. This technique allows for improved control of confounding in observational data and is particularly effective when used in conjunction with weighted regression models [22,23]. Covariates included age, sex, race/ethnicity, Medicaid coverage, Elixhauser Comorbidity Index, and specific psychiatric diagnoses (ADHD, schizophrenia, bipolar disorder) [25]. Covariate balance was assessed using standardized differences, with a threshold of <0.1 indicating acceptable balance. After weighting, all variables met this criterion, suggesting successful balancing (Supplementary Materials, Table A1, Table A2, Table A3 and Table A4).

Weighted logistic regression models were used to estimate the association between COVID-19 infection and the incidence of depression and anxiety, adjusting for the entropy-balanced covariates. To evaluate the robustness of these associations to unmeasured confounding, E-values were calculated for the COVID-19 infection variable in each model [26]. All analyses were conducted using Stata version 18 (College Station, TX, USA), with a two-sided significance threshold set at *p* < 0.05.

## 3. Results

### 3.1. Incidence of Depression

A total of 462,537 adults had continuous insurance coverage from 2019 through 2021. After excluding 105,552 individuals with a depression diagnosis in 2019 or 2020 or those dually eligible for Medicaid and Medicare, the final sample included 356,985 adults. The mean age in 2019 was 39 years (SD = 17), and 48% were female. Approximately 38% had a documented COVID-19 infection in 2020.

The incidence of new depression diagnoses in 2021 was significantly higher among individuals with prior COVID-19 infection compared to those without (7.47% vs. 4.52%; *p* < 0.01) (Table 1).

In the adjusted model, individuals with a COVID-19 infection in 2020 had 48% higher odds of receiving a depression diagnosis in 2021 (OR = 1.48; *p* < 0.01). Age was a significant predictor of new depression diagnoses. Compared to adults aged 51–62, younger individuals had higher odds: ages 18–30 (OR = 1.91; *p* < 0.01), 31–40 (OR = 1.45; *p* < 0.01), and 41–50 (OR = 1.20; *p* < 0.01). Females had substantially higher odds of depression compared to males (OR = 1.87; *p* < 0.01). Although individuals with Medicaid coverage showed a modest increase in the odds of depression (OR = 1.05), the association was not statistically significant (*p* = 0.12). A higher comorbidity severity score was associated with increased odds of depression (OR = 1.03; *p* < 0.01). Additionally, specific conditions—pain-related diagnoses (OR = 1.24; *p* < 0.01), ADHD (OR = 1.65; *p* < 0.01), and substance use disorder (OR = 1.92; *p* < 0.01)—were significantly associated with new depression diagnoses in 2021 (Table 2).

To assess the potential impact of unmeasured confounding on the observed association between COVID-19 infection and incident depression, we calculated the E-value. E-value quantifies the minimum strength of association an unmeasured confounder would require with both exposure and outcome to fully explain away the observed association [27,28]. In our analysis, the point estimate for the E-value was 2.31, which exceeds the observed odds ratio for COVID-19 infection (OR = 1.47) and all other measured covariate in the model. This indicates that an unmeasured confounder would need to be strongly associated with both COVID-19 infection and new depression diagnoses by a risk ratio of at least 2.31, independent of other measured factors to negate our findings.

### 3.2. Incidence of Anxiety

From the initial cohort of 462,537 adults continuously enrolled in either Medicaid or private insurance from 2019 through 2021, individuals dually eligible for Medicare and those with an anxiety diagnosis recorded in 2019 or 2020 were excluded (n = 91,046), yielding a final analytic sample of 371,491 adults. Among this population, 38% had a documented COVID-19 infection in 2020. In 2021, the incidence of new anxiety diagnoses was 4.70% among individuals without a COVID-19 infection, compared to 7.73% among those who had been infected (*p* < 0.01) (Table 3).

Multivariable regression results indicated that individuals with a COVID-19 infection in 2020 had a 46% higher likelihood of receiving an anxiety diagnosis in 2021 (OR = 1.46, *p* < 0.01). Female subjects exhibited a significantly elevated risk compared to males (OR = 1.77, *p* < 0.01), and Medicaid coverage was associated with a modest but statistically significant increase in anxiety risk (OR = 1.07, *p* < 0.01). Pre-existing mental health conditions in 2019, such as ADHD (OR = 1.22, *p* < 0.01) and cognitive disorders (OR = 1.32, *p* < 0.01), were also associated with increased likelihood of an anxiety diagnosis in 2021. Additionally, physical health conditions including obesity (OR = 1.31, *p* < 0.01) and thyroid disorders (OR = 1.16, *p* < 0.01) were significantly associated with anxiety incidence (Table 4).

The resulting E-value of 2.29 exceeded the odds ratio for COVID-19 infection and all other covariates in the model, suggesting that unmeasured confounding would need to be associated with both the exposure and the outcome by a risk ratio of at least 2.29 to nullify the observed association.

## 4. Discussion

In this large, population-based study using the Utah All Payers Claims Database, adults with a documented COVID-19 infection in 2020 were significantly more likely to receive new diagnoses of depression and anxiety in 2021 compared to those without infection. These associations remained robust after adjustment for demographic characteristics, comorbidity burden, Medicaid coverage, and pre-existing psychiatric conditions. Sensitivity analyses using E-values indicated that substantial unmeasured confounding would be required to fully explain away these associations, supporting the validity of the findings.

Our findings align with prior studies demonstrating an increased risk of mental health disorders following COVID-19 infection. A case-control study by Zhang et al., reported a higher incidence of depression (29.2%) among individuals who contracted COVID-19 compared to those in quarantine or the general population [27]. Similarly, a study of 714 COVID-19 patients found that 96.2% experienced posttraumatic stress symptoms associated with the infection [18]. In another study, Mazza et al. assessed psychiatric outcomes in over 400 adults recovering from COVID-19 and found that 31% had clinical depression, while 42% were diagnosed with anxiety [19]. These patterns suggest that the psychiatric sequelae of COVID-19 extend beyond acute illness, reflecting a combination of biological vulnerability, psychosocial stressors, and gaps in mental health support during recovery.

Consistent with previous literature, we found that women were substantially more likely to be diagnosed with depression following COVID-19 infection [29]. This gender disparity is well documented in mental health research, with women generally facing higher lifetime risks of depression and anxiety [7,16,30,31]. The pandemic may have amplified this risk through increased caregiving responsibilities, job loss, and financial strain [32]. Biological mechanisms may also play a role. For instance, inflammatory responses to COVID-19 infection may interact with sex-based differences in immune function. Eisenberger and colleagues have shown that women may be more susceptible to dysphoric reactions linked to inflammation, underscoring the complex interplay between biological and psychosocial determinants of mental health [16].

We also observed elevated risk among younger adults, who were more likely than older age groups to be diagnosed with depression after infection. This finding is consistent with studies by Mazza et al. and Wang et al., which reported higher rates of depressive symptoms among younger individuals during the pandemic [7,33]. Although causality cannot be inferred, several plausible explanations exist. Younger individuals faced unique disruptions such as school closures, interruptions to academic and career plans, reduced social interaction, economic uncertainty, and heightened fear of infection that may have intensified psychological distress [33].

Furthermore, individuals covered by Medicaid—a proxy for lower socioeconomic status were 3% more likely to receive a depression diagnosis compared to those with private insurance. While the effect size was modest, the direction of the association is also aligns with prior studies showing that lower-income and socially disadvantaged populations were disproportionately affected by pandemic-related stressors and exhibited higher rates of mental health disorders [33].

This study has several limitations. First, the database did not record the severity of COVID-19 symptoms, which could potentially influence the association between COVID-19 infection and the incidence of depression and anxiety. However, the primary focus of the study was to examine how COVID-19 infection could be associated with the incidence of depression or anxiety. Second, the dataset captured only medically confirmed cases of COVID-19 i.e., those for which individuals sought clinical care. Asymptomatic cases or individuals who self-managed without formal diagnosis were excluded, potentially leading to underestimation of COVID-19 exposure and attenuation of the observed association. Furthermore, although individuals with documented depression or anxiety diagnoses in 2019 and 2020 were excluded, undiagnosed or subclinical cases may have been missed due to inconsistent or limited mental health screening practices in primary care settings. This potential under-detection could lead to misclassification bias, resulting in an overestimation of incident cases in 2021. We acknowledge this limitation may affect the precision of our findings and highlight the need for cautious interpretation. Third, the generalizability of the findings is limited. The study population was restricted to adults aged 18 to 64 years, excluding children and elderly individuals who may experience different mental health trajectories following infection. Furthermore, a substantial proportion of individuals in the dataset have missing race and ethnicity information, hindering the ability to fully explore disparities in mental health outcomes across demographic groups. Finally, we did not include data on pharmacologic treatment. Diagnoses were identified using ICD-10 codes, without linked prescription information. Some individuals may have received mental health treatment without a recorded diagnosis, or vice versa, leading to further potential misclassification.

Despite these limitations, our findings have important implications for public health practice. The increased risk of depression and anxiety following COVID-19 infection underscores the need for targeted mental health interventions [34,35,36].

Public awareness campaigns should highlight the potential mental health consequences of COVID-19, normalize help-seeking behavior, and promote available support resources. Additionally, training healthcare professionals to identify and manage psychiatric symptoms among COVID-19 patients particularly in primary care and acute care settings may help close existing gaps in mental health detection and treatment.

Efforts to enhance access to mental health services must be prioritized, especially for vulnerable groups such as women, economically disadvantaged individuals, and those with limited insurance coverage. Policy measures aimed at broadening Medicaid eligibility and improving coverage for mental health services are critical. In sum, addressing the mental health aftermath of the COVID-19 pandemic requires coordinated public health strategies and a deliberate focus on equity in mental health care access and delivery.

## 5. Conclusions

This study highlighted the increased risk of depression and anxiety among individuals infected with COVID-19, emphasizing the importance of addressing the mental health needs of those affected by the virus. By leveraging population-level claims data and applying rigorous statistical methods, our findings provide robust evidence of incident psychiatric conditions following infection. These results contribute valuable insight into the long-term impacts of COVID-19 and support the integration of mental health care into post-infection recovery efforts.

## Figures and Tables

**Table 1:** Characteristics of subjects with and without a COVID-19 infection in 2020 regarding the incidence of depression outcomes.

		COVID-19 Infection in 2020		*p*-Value
Variable	Overall (n = 356,985; 100%)	No (n = 222,642; 62.37%)	Yes (n = 134,343; 37.63%)	
	Mean (SD)/%	Mean (SD)/%	Mean (SD)/%	
Age (as continuous)	39.38 (16.90)	39.37 (15.03)	39.39 (18.14)	0.62
Age category (%)				0.99
18–30	28.80	28.80	28.80	
31–40	24.30	24.30	24.30	
41–50	23.20	23.20	23.20	
51–62	23.70	23.70	23.70	
Female (%)	51.90	51.90	51.90	0.96
Race/Ethnicity (%)				
Non-Hispanic White	12.20	12.20	12.20	0.96
Non-Hispanic Black	0.40	0.40	0.40	
Non-Hispanic Asian/Pacific Islander/American Indian	1.00	1.00	1.00	
Hispanic	2.10	2.10	2.10	
Unknown	84.30	84.30	84.30	
Medicaid coverage (%)	5.90	5.90	5.90	0.82
Elixhauser comorbidity index score	0.63 (1.43)	0.63 (1.43)	0.63 (1.43)	1.00
Comorbid condition (%)				
Anxiety	7.30	7.30	7.30	0.99
Schizophrenia	0.20	0.20	0.20	0.82
Bipolar	0.10	0.10	0.10	0.99
Cognitive disorder	2.70	2.70	2.70	0.99
ADHD	2.20	2.20	2.20	0.99
Sleep disorder	5.40	5.40	5.40	0.99
Nutritional deficiency/malnutrition	1.90	1.90	1.90	0.99
Pain	31.63	31.63	31.70	0.96
Diabetes	4.50	4.50	4.50	0.99
Hypertension	11.40	11.40	11.40	0.99
Obesity	16.70	16.70	16.70	0.98
Low back pain	20.80	20.80	20.80	0.97
Asthma/COPD	2.50	2.50	2.50	0.99
Substance use disorder	1.10	1.10	1.10	0.98
Incidence of depression in 2021 (%)	5.63	4.52	7.47	<0.01

Note: ADHD = attention-deficit/hyperactivity disorder, COPD = chronic obstructive pulmonary disease.

**Table 2:** Factors associated with the incidence of depression through weighted logistic regression.

Covariate	OR	*p*-Value	95% Confidence Interval	
COVID-19 infection	1.48	<0.01	1.43	1.52
Age category				
18–30	1.91	<0.01	1.82	2.00
31–40	1.45	<0.01	1.38	1.52
41–50	1.20	<0.01	1.14	1.26
51–62	reference			
Female	1.87	<0.01	1.81	1.93
Race/Ethnicity				
Non-Hispanic White	reference			
Non-Hispanic Black	0.75	0.02	0.58	0.95
Non-Hispanic Asian/Pacific Islander/American Indian	0.56	<0.01	0.47	0.67
Hispanic	0.84	<0.01	0.76	0.93
Unknown	0.81	<0.01	0.77	0.85
Medicaid coverage	1.05	0.12	0.99	1.12
Elixhauser comorbidity index score	1.03	0.01	1.01	1.05
Anxiety	2.33	<0.01	2.23	2.44
Schizophrenia	1.25	0.07	0.98	1.59
Bipolar	1.25	0.16	0.92	1.71
Cognitive disorder	0.95	0.41	0.84	1.07
ADHD	1.65	<0.01	1.46	1.88
Sleep disorder	1.32	<0.01	1.24	1.42
Nutritional deficiency/malnutrition	1.05	0.38	0.94	1.17
Pain	1.24	<0.01	1.19	1.31
Diabetes	1.05	0.30	0.96	1.13
Hypertension	1.04	0.28	0.97	1.10
Obesity	1.66	<0.01	1.59	1.73
Low back pain	1.04	0.19	0.98	1.10
Asthma/COPD	1.15	<0.01	1.05	1.27
Substance use disorder	1.92	<0.01	1.72	2.15

Note: ADHD = attention-deficit/hyperactivity disorder, COPD = chronic obstructive pulmonary disease.

**Table 3:** Characteristics of subjects with and without a COVID-19 infection in 2020 regarding the incidence of anxiety outcomes.

		COVID-19 Infection in 2020		*p*-Value
Variable	Overall (n = 371,491)	No (n = 229,990; 61.91%)	Yes (n = 141,501; 38.09%)	
	Mean (SD)/%	Mean (SD)/%	Mean (SD)/%	
Age (as continuous)	39.67 (16.54)	39.96 (15.42)	39.98 (18.61)	0.65
Age category (%)				1.00
18–30	27.43	27.43	27.43	
31–40	23.59	23.59	23.59	
41–50	23.41	23.41	23.41	
51–62	25.58	25.58	25.58	
Female (%)	52.99	52.99	52.98	0.95
Race/Ethnicity (%)				0.98
Non-Hispanic White	13.35	13.35	13.36	
Non-Hispanic Black	0.36	0.36	0.36	
Non-Hispanic Asian/Pacific Islander/American Indian	1.00	1.00	1.00	
Hispanic	2.19	2.19	2.19	
Unknown	83.10	83.10	83.09	
Medicaid coverage (%)	7.01	7.01	7.03	0.82
Elixhauser comorbidity index score	0.85 (2.25)	0.85 (2.33)	0.85 (2.12)	0.99
Comorbid condition (%)				
Depression	11.50	11.50	11.50	0.99
Schizophrenia	0.27	0.27	0.27	0.87
Bipolar	0.80	0.80	0.80	0.99
Cognitive disorder	2.79	2.79	2.79	0.94
ADHD	2.29	2.29	2.29	0.99
Sleep disorder	6.31	6.31	6.31	0.99
Nutritional deficiency/malnutrition	2.16	2.16	2.16	0.99
Pain	33.08	33.08	33.07	0.95
Diabetes	5.50	5.50	5.50	0.99
Hypertension	12.94	12.94	12.94	0.99
Obesity	19.06	19.06	19.05	0.98
Low back pain	22.15	22.15	22.14	0.97
Asthma/COPD	2.76	2.76	2.76	0.99
Substance use disorder	1.63	1.63	1.63	0.99
IDD	0.63	0.63	0.63	0.78
Thyroid disorder	5.48	5.48	5.48	0.99
Menopausal disorder	3.81	3.81	3.81	0.99
Chronic kidney disease	0.63	0.63	0.63	1.00
Incidence of anxiety in 2021 (%)	5.85	7.73	4.69	<0.01

Note: ADHD = attention-deficit/hyperactivity disorder, COPD = chronic obstructive pulmonary disease, IDD = intellectual and developmental disabilities.

**Table 4:** Factors associated with the incidence of anxiety through weighted logistic regression.

Covariate	Odds Ratio (OR)	*p*-Value	95% Confidence Interval	
COVID-19 infection	1.46	<0.01	1.41	1.50
Age category				
18–30	2.45	<0.01	2.34	2.56
31–40	1.77	<0.01	1.69	1.86
41–50	1.41	<0.01	1.35	1.48
51–62	reference			
Female	1.77	<0.01	1.72	1.83
Race/Ethnicity				
Non-Hispanic White	reference			
Non-Hispanic Black	0.52	<0.01	0.40	0.68
Non-Hispanic Asian/Pacific Islander/American Indian	0.50	<0.01	0.42	0.60
Hispanic	0.78	<0.01	0.70	0.86
Unknown	0.84	<0.01	0.80	0.87
Medicaid coverage	1.08	0.01	1.03	1.14
Elixhauser comorbidity index score	1.01	0.12	1.00	1.03
Anxiety	2.24	<0.01	2.14	2.34
Schizophrenia	1.43	<0.01	1.17	1.76
Bipolar	1.11	0.08	0.99	1.26
Cognitive disorder	1.32	<0.01	1.16	1.51
ADHD	1.22	<0.01	1.07	1.40
Sleep disorder	1.22	<0.01	1.14	1.30
Nutritional deficiency/malnutrition	1.12	0.02	1.02	1.23
Pain	1.36	<0.01	1.30	1.42
Diabetes	0.79	<0.01	0.72	0.85
Hypertension	0.96	0.21	0.91	1.02
Obesity	1.31	<0.01	1.26	1.37
Low back pain	0.96	0.11	0.91	1.01
Asthma/COPD	1.07	0.14	0.98	1.17
Substance use disorder	1.49	<0.01	1.34	1.65
IDD	0.70	<0.01	0.58	0.85
Thyroid disorder	1.16	<0.01	1.09	1.23
Menopausal disorder	1.16	<0.01	1.08	1.24
Chronic kidney disease	0.81	0.06	0.65	1.01

Note: ADHD = attention-deficit/hyperactivity disorder, COPD = chronic obstructive pulmonary disease, IDD = intellectual and developmental disabilities.

## Data Availability

Data cannot be shared publicly because of the regulations of Utah Department of Health and Human Services, and the Utah Resource for Genetic and Epidemiologic Research (RGE). Data are available from the University of Utah Institutional Data Access/Ethics Committee (contact via RGE: rge@hsc.utah.edu) for researchers who meet the criteria for access to confidential data.

## Supplementary Materials

The following supporting information can be downloaded at: https://www.mdpi.com/article/10.3390/healthcare13141638/s1.

## Appendix A

**Table A1:** Standardized mean differences before and after entropy balancing for the occurrence of depression.

	Before Entropy Balancing						
	COVID-19 Infection			No COVID-19 Infection			Standardized Difference
Variable	Mean	Variance	Skewness	Mean	Variance	Skewness	
Age category							
Age 18 to 30	0.288	0.205	0.937	0.283	0.203	0.964	0.011
Age 31 to 40	0.243	0.184	1.201	0.245	0.185	1.187	−0.005
Age 41 to 50	0.232	0.178	1.272	0.233	0.179	1.264	−0.003
Age 51 to 62	reference						
Female	0.519	0.250	−0.076	0.452	0.248	0.192	0.134
Race/Ethnicity							
Non-Hispanic White	reference						
Non-Hispanic Black	0.003	0.003	16.890	0.004	0.004	15.400	−0.012
Non-Hispanic Asian/Pacific Islander/American Indian	0.010	0.010	10.000	0.012	0.011	9.123	−0.019
Hispanic	0.021	0.021	6.681	0.019	0.019	7.030	0.013
Unknown	0.843	0.132	−1.889	0.822	0.146	−1.682	0.059
Medicaid coverage	0.059	0.056	3.740	0.097	0.087	2.728	−0.160
Elixhauser comorbidity index score	0.625	1.430	3.097	0.472	1.062	3.444	0.128
Anxiety	0.073	0.068	3.279	0.050	0.047	4.134	0.089
Schizophrenia	0.002	0.002	21.760	0.005	0.005	13.760	−0.068
Bipolar	0.001	0.001	27.810	0.001	0.001	26.610	−0.003
Cognitive disorder	0.027	0.027	5.793	0.027	0.026	5.863	0.004
ADHD	0.022	0.022	6.508	0.016	0.016	7.655	0.040
Sleep disorder	0.054	0.051	3.929	0.035	0.034	5.060	0.086
Nutritional deficiency/malnutrition	0.019	0.019	7.032	0.012	0.012	8.954	0.052
Pain	0.317	0.216	0.789	0.217	0.170	1.377	0.215
Diabetes	0.045	0.043	4.390	0.038	0.037	4.835	0.034
Hypertension	0.114	0.101	2.432	0.095	0.086	2.756	0.058
Obesity	0.167	0.139	1.786	0.113	0.100	2.448	0.145
Low back pain	0.208	0.165	1.440	0.141	0.121	2.059	0.164
Asthma/COPD	0.025	0.024	6.148	0.016	0.016	7.743	0.056
Substance use disorder	0.011	0.011	9.231	0.011	0.011	9.182	−0.001

**Table A2:** Standardized mean differences before and after entropy balancing for the occurrence of depression (continued).

	After Entropy Balancing						
	COVID-19 Infection			No COVID-19 Infection			Standardized Difference
Variable	Mean	Variance	Skewness	Mean	Variance	Skewness	
Age category							
Age 18 to 30	0.288	0.205	0.937	0.288	0.205	0.937	0.000
Age 31 to 40	0.243	0.184	1.201	0.243	0.184	1.201	0.000
Age 41 to 50	0.232	0.178	1.272	0.232	0.178	1.272	0.000
Age 51 to 62	reference						
Female	0.519	0.250	−0.076	0.519	0.250	−0.076	0.000
Race/Ethnicity							
Non-Hispanic White	reference						
Non-Hispanic Black	0.003	0.003	16.890	0.003	0.003	16.880	0.000
Non-Hispanic Asian/Pacific Islander/American Indian	0.010	0.010	10.000	0.010	0.010	9.998	0.000
Hispanic	0.021	0.021	6.681	0.021	0.021	6.680	0.000
Unknown	0.843	0.132	−1.889	0.843	0.132	−1.888	0.000
Medicaid coverage	0.059	0.056	3.740	0.059	0.056	3.733	−0.001
Elixhauser comorbidity index score	0.625	1.430	3.097	0.625	1.430	3.097	0.000
Anxiety	0.073	0.068	3.279	0.073	0.068	3.279	0.000
Schizophrenia	0.002	0.002	21.760	0.002	0.002	21.580	−0.001
Bipolar	0.001	0.001	27.810	0.001	0.001	27.790	0.000
Cognitive disorder	0.027	0.027	5.793	0.027	0.027	5.791	0.000
ADHD	0.022	0.022	6.508	0.022	0.022	6.509	0.000
Sleep disorder	0.054	0.051	3.929	0.054	0.051	3.930	0.000
Nutritional deficiency/malnutrition	0.019	0.019	7.032	0.019	0.019	7.033	0.000
Pain	0.317	0.216	0.789	0.316	0.216	0.790	0.000
Diabetes	0.045	0.043	4.390	0.045	0.043	4.391	0.000
Hypertension	0.114	0.101	2.432	0.114	0.101	2.432	0.000
Obesity	0.167	0.139	1.786	0.167	0.139	1.787	0.000
Low back pain	0.208	0.165	1.440	0.208	0.165	1.440	0.000
Asthma/COPD	0.025	0.024	6.148	0.025	0.024	6.148	0.000
Substance use disorder	0.011	0.011	9.231	0.011	0.011	9.226	0.000

## Appendix B

**Table A3:** Standardized mean differences before and after entropy balancing for the occurrence of anxiety.

	Before Entropy Balancing						
	COVID-19 Infection			No COVID-19 Infection			Standardized Difference
Variable	Mean	Variance	Skewness	Mean	Variance	Skewness	
Age category							
Age 18 to 30	0.274	0.199	1.012	0.275	0.199	1.009	−0.001
Age 31 to 40	0.236	0.180	1.244	0.240	0.182	1.218	−0.010
Age 41 to 50	0.234	0.179	1.256	0.234	0.179	1.255	0.000
Age 51 to 62	reference						
Female	0.530	0.249	−0.120	0.459	0.248	0.166	0.143
Race/Ethnicity							
Non-Hispanic White	reference						
Non-Hispanic Black	0.004	0.004	16.680	0.004	0.004	14.920	−0.015
Non-Hispanic Asian/Pacific Islander/American Indian	0.010	0.010	9.842	0.012	0.012	9.076	−0.017
Hispanic	0.022	0.021	6.535	0.020	0.020	6.865	0.013
Unknown	0.831	0.140	−1.767	0.811	0.153	−1.592	0.053
Medicaid coverage	0.070	0.065	3.369	0.107	0.095	2.551	−0.143
Elixhauser comorbidity index score	0.852	2.239	2.955	0.620	1.569	3.221	0.155
Depression	0.115	0.102	2.414	0.079	0.073	3.127	0.114
Schizophrenia	0.003	0.003	19.140	0.006	0.006	13.130	−0.058
Bipolar	0.008	0.008	11.060	0.007	0.007	11.480	0.006
Cognitive disorder	0.028	0.027	5.736	0.027	0.026	5.843	0.006
ADHD	0.023	0.022	6.384	0.017	0.017	7.434	0.038
Sleep disorder	0.063	0.059	3.594	0.040	0.039	4.687	0.094
Nutritional deficiency/malnutrition	0.022	0.021	6.576	0.014	0.013	8.373	0.055
Pain	0.331	0.221	0.719	0.227	0.175	1.304	0.221
Diabetes	0.055	0.052	3.906	0.045	0.043	4.408	0.045
Hypertension	0.129	0.113	2.208	0.107	0.095	2.546	0.067
Obesity	0.191	0.154	1.576	0.126	0.110	2.253	0.164
Low back pain	0.222	0.172	1.341	0.150	0.128	1.957	0.171
Asthma/COPD	0.028	0.027	5.764	0.018	0.017	7.317	0.061
Substance use disorder	0.016	0.016	7.642	0.015	0.015	7.958	0.010
IDD	0.006	0.006	12.520	0.014	0.014	8.335	−0.096
Thyroid disorder	0.055	0.052	3.912	0.038	0.037	4.821	0.073
Menopausal disorder	0.038	0.037	4.827	0.023	0.022	6.432	0.081
Chronic kidney disease	0.006	0.006	12.500	0.004	0.004	15.000	0.024

**Table A4:** Standardized mean differences before and after entropy balancing for the occurrence of anxiety (continued).

	After Entropy Balancing						
	COVID-19 Infection			No COVID-19 Infection			Standardized Difference
Variable	Mean	Variance	Skewness	Mean	Variance	Skewness	
Age category							
Age 18 to 30	0.274	0.199	1.012	0.274	0.199	1.012	0.000
Age 31 to 40	0.236	0.180	1.244	0.236	0.180	1.244	0.000
Age 41 to 50	0.234	0.179	1.256	0.234	0.179	1.256	0.000
Age 51 to 62	reference						
Female	0.530	0.249	−0.120	0.530	0.249	−0.120	0.000
Race/Ethnicity							
Non-Hispanic White	reference						
Non-Hispanic Black	0.004	0.004	16.680	0.004	0.004	16.670	0.000
Non-Hispanic Asian/Pacific Islander/American Indian	0.010	0.010	9.842	0.010	0.010	9.839	0.000
Hispanic	0.022	0.021	6.535	0.022	0.021	6.534	0.000
Unknown	0.831	0.140	−1.767	0.831	0.141	−1.766	0.000
Medicaid coverage	0.070	0.065	3.369	0.070	0.065	3.363	−0.001
Elixhauser comorbidity index score	0.852	2.239	2.955	0.852	2.239	2.955	0.000
Depression	0.115	0.102	2.414	0.115	0.102	2.414	0.000
Schizophrenia	0.003	0.003	19.140	0.003	0.003	19.040	−0.001
Bipolar	0.008	0.008	11.060	0.008	0.008	11.050	0.000
Cognitive disorder	0.028	0.027	5.736	0.028	0.027	5.731	0.000
ADHD	0.023	0.022	6.384	0.023	0.022	6.385	0.000
Sleep disorder	0.063	0.059	3.594	0.063	0.059	3.595	0.000
Nutritional deficiency/malnutrition	0.022	0.021	6.576	0.022	0.021	6.577	0.000
Pain	0.331	0.221	0.719	0.331	0.221	0.720	0.000
Diabetes	0.055	0.052	3.906	0.055	0.052	3.906	0.000
Hypertension	0.129	0.113	2.208	0.129	0.113	2.208	0.000
Obesity	0.191	0.154	1.576	0.191	0.154	1.576	0.000
Low back pain	0.222	0.172	1.341	0.221	0.172	1.342	0.000
Asthma/COPD	0.028	0.027	5.764	0.028	0.027	5.764	0.000
Substance use disorder	0.016	0.016	7.642	0.016	0.016	7.640	0.000
IDD	0.006	0.006	12.520	0.006	0.006	12.450	−0.001
Thyroid disorder	0.055	0.052	3.912	0.055	0.052	3.912	0.000
Menopausal disorder	0.038	0.037	4.827	0.038	0.037	4.828	0.000
Chronic kidney disease	0.006	0.006	12.500	0.006	0.006	12.500	0.000
